# Peak occipital alpha frequency mediates the relationship between sporting expertise and multiple object tracking performance

**DOI:** 10.1002/brb3.3434

**Published:** 2024-02-21

**Authors:** Andrew K. Mackenzie, Joshua Baker, Rosie C. Daly, Christina J. Howard

**Affiliations:** ^1^ NTU Psychology Nottingham Trent University Nottingham UK; ^2^ Department of Psychology University of Essex Colchester UK

**Keywords:** cognition, electroencephalography, psychology

## Abstract

**Background:**

Multiple object tracking (MOT) is often used as a lab‐based paradigm for investigating goal‐driven attention as an indicator for “real‐world” attention in tasks such as sport. When exploring MOT performance in the context of sporting expertise, we typically observe that individuals with sporting expertise outperform non‐sporting individuals. There are a number of general explanations for performance differences such as cognitive transfer effects; however, the potential neurophysiological mechanisms explaining the relationship between sporting expertise and performance differences in MOT are not clear. Based on the role occipital alpha (posterior oscillations usually around 8–12 Hz) has been shown to have in visuospatial attention, the aim of this study was to examine whether individual differences in occipital peak alpha frequency (PAF) mediate the relationship between sporting expertise and performance in two object tracking tasks: a standard MOT task and a visuomotor‐controlled object tracking task (multiple object avoidance [MOA]).

**Method:**

Using electroencephalography (EEG), participants, who either played sport competitively or did not, had their posterior PAF measured at rest (eyes closed) across a 2‐min window. They completed the two tasks separately from the resting EEG measures.

**Results:**

Those who engaged in sport performed better in the MOT and MOA tasks and had higher PAF. Higher PAF predicted superior MOT performance. The mediation analysis revealed that sporting individuals had significantly higher PAF, and this was in turn related to superior MOT performance.

**Conclusions:**

It is suggested that PAF is a possible neurophysiological mediating mechanism as to why sporting individuals have superior MOT performance. There was no evidence that PAF mediated the relationship between sporting expertise and visuomotor MOA performance. Explanations and implications are discussed, and unanswered questions are proposed.

## Graphical abstract

Occipital peak alpha frequency mediates the relationship between sporting expertise and multiple object tracking performance.

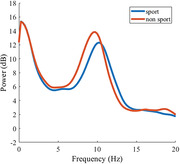

## 1 INTRODUCTION

Performance in sport requires the engagement of several cognitive processes. Sport likely requires, for example, selective attention, inhibition of task‐irrelevant information, working memory function, planning ability, and, importantly for this current study, sustained divided attention to multiple items. For example, in football (soccer), an individual who is about to receive an intended pass may have to scan the field to identify to whom they may in‐turn pass, inhibit irrelevant opposition players, and divide their attention between the ball position, any relevant same‐team players’ position, and any relevant opposing‐team players’ position. There are a number of cognitive tasks that aim to measure this complex goal‐driven attention in dynamic environments. Arguably, attention ability required in many sports is akin to a multiple object tracking (MOT) paradigm (Faubert, 2013; Mackenzie et al., 2022; Meyerhoff et al., 2017). In a typical MOT paradigm, an individual is tasked with attending to a set number of dynamic objects while ignoring distractor objects. As all the objects move, the individual must sustain their attention on the target objects while ignoring the distractor objects and then, after movement stops, select the original targets (Cavanagh & Alvarez, 2005; Pylyshyn & Storm, 1988). Given the nature of the MOT as being an attentionally demanding task requiring elements of selective and sustained attention (Drew & Vogel, 2008; Meyerhoff et al., 2017), it has often been used as a paradigm for investigating goal‐driven attention in the lab often as an indicator for real‐world task‐based attention (Mackenzie et al., 2022). When exploring MOT performance in the context of sporting expertise, we observe findings typical across other cognitive tasks. That is, individuals with sporting expertise typically outperform non‐sporting individuals (Harris et al., 2020; Howard et al., 2018; Mackenzie et al., 2022; Qiu et al., 2018); for example, Howard et al. (2018) showed that team ball sport participation was associated with superior localization for tracked objects. There are a number of general explanations for performance differences that fit with the sporting literature such as transfer effects of training and expertise (e.g., Allen et al., 2004); however, the underlying mechanisms explaining the relationship between sporting expertise and performance differences in MOT are not clear. In this study, we aimed to explore a potential neurophysiological basis of MOT performance in those who partake in sport. We first discuss the behavioral and cognitive relationship between sporting expertise and MOT performance before discussing the possible neurophysiological mechanism that might mediate this relationship.

### 1.1 Attention and object tracking expertise in sport

A high level of attentional ability is important in sporting success (Janelle & Hatfield, 2008; Memmert, 2009), but equally and inversely, we find that sporting expertise positively predicts performance in attention tasks (e.g., Tomporowski & Pesce, 2019; Vestberg et al., 2012; Voss et al., 2010). One might predict there could be an enhancement in general attentional ability (e.g., tracking) due to playing attentionally demanding sport. In one of the few early pieces of research exploring sporting expertise and attentional ability, Memmert (2006) investigated how sporting expertise might link to inattentional blindness. They used the Simons and Chabris (1999) inattentional blindness paradigm where participants view a video and track and count the number of basketball passes between a number of individuals but are later asked if they identified a Gorilla during the visual scene. Given the assumed higher level of attentional ability due to having basketball‐specific‐related expertise, they predicted that expert basketball players would be more likely to detect the gorilla than novice basketball players. This is what the authors found (Memmert, 2006). The theoretical implications of this early research could be that experts were better able to distribute spatial attention and/or better divide (fewer) attentional resources to track the balls thus allowing one to also identify the gorilla. This raises the question of whether the superior attentional performance was context (basketball) specific or if sporting experts have a more generally superior attentional ability.

In follow‐up research related to MOT, Memmert et al. (2009) examined domain‐non‐specific attention differences between different levels of sporting expertise using an MOT task. They compared team sports athletes, individual sports athletes, and sporting novices. Unexpectedly, performance was similar across sporting groups. This could be interpreted to suggest no enhancement in general attentional or, specifically, MOT ability due to sporting expertise. However, perhaps these earlier results are not too surprising. Certainly, in the two athlete groups, all participants engaged in sporting and exercise activity, and even the novice athlete condition was largely non‐restrictive to those who have no more than 2 years of experience in sport. There is evidence to suggest the cognitive and attentional benefit in simply engaging in sport more generally (e.g., Tomporowski & Pesce, 2019), and therefore it is possible then that there is a superiority effect in MOT ability, but this is observed more clearly between those who engage in sports and those who do not, rather than across expertise within a sporting discipline. This has since been found in a number of studies (Harris et al., 2020; Howard et al., 2018; Mackenzie et al., 2022; Qiu et al., 2018) where sporting individuals outperform non‐sporting individuals in MOT. This corroborates evidence for links between sports playing and cognitive function, and also in non‐sporting but other real‐world expertise in object tracking such as in radar operation (e.g., Allen et al., 2004) and videogames (e.g., Green & Bavelier, 2006) where real‐world task expertise predicts superior MOT performance.

### 1.2 Neurophysiological explanations of MOT performance

Theoretical explanations for the superiority in MOT performance in those with sporting expertise (or experience) often focus on the effects of cognitive transfer due to practice or training similar attentional abilities in sports as in MOT (Bier et al., 2018; Dahlin et al., 2008; Harris et al., 2020; Peng & Miller, 2016; Posner et al., 2015). Arguably, although important and informative, these explanations may be considered more general and still may not offer truly target *why* there is, for example, cognitive transfer. We can explore possible neurophysiological evidence for this link. There have been a number of event‐related potentials linked to MOT performance. For example, Drew et al. (2009) reported that larger N1 magnitudes, which are early visually evoked responses sensitive to the allocation of spatial attention, relate to better tracking performance in an MOT. In addition, Drew and Vogel (2008) explored the role of N2pc, which reflects the selection of visual stimuli among distractors (Hopf, 2000; Luck et al., 1997), and posterior contralateral delay activity (CDA) which, linked to sustained attention, appears sensitive to the storage of objects in visual working memory (Vogel & Machizawa, 2004; Vogel et al., 2005). They report that amplitudes in both N2pc and CDA activity dropped as a function of a number of objects to track (i.e. 3–5) in individuals with poor tracking but not in those who were considered good trackers. In other words, good trackers were able to maintain the level of attention during harder tracking trials. However, these may not provide clear indicators as to why sporting individuals might perform better at MOT as it is unclear how these components are observed generally in sports players.

Neural oscillations at the alpha frequency (8–12 Hz) across the brain have been linked to cognitive performance and ability in a number of ways, with higher alpha power generally linked to inhibition (Klimesch et al., 2007). Furthermore, individual differences in the peak spectral frequency of alpha activity have been linked with cognitive processing speed, with faster alpha/higher frequency associated with better cognitive abilities (Angelakis et al., 2004; Klimesch, 1999, though see Posthuma et al., 2001) and alpha frequency slowing with age (Klimesch, 1999; Scally et al., 2018). According to the perceptual cycles hypothesis of brain function, at posterior sites where visual processing occurs, it has been argued that alpha oscillations reflect discrete temporal sampling in vision (Dugué et al., 2011; VanRullen, 2016) whereby periodic phases of visual inhibition are followed by periods of visual sampling. This is supported by evidence that transcranial magnetic stimulation‐ (TMS) induced visual phosphenes depend on the phase of the ongoing alpha activity (Fakche et al., 2022) and that the phase of pre‐stimulus alpha appears to predict whether two stimuli occurring close in time are perceived as synchronous or asynchronous (Milton & Pleydell‐Pearce, 2016). This idea is further supported by evidence that the visual system appears to sample information at an alpha frequency (Crouzet & VanRullen, 2017), but that this sampling appears to halve in frequency when attention is divided between two stimuli, suggesting serial switching of this ‘flickering spotlight’ of visual attention.

The characteristics of alpha oscillations also appear to respond to task demands. For example, Samuel et al. (2018) showed that alpha frequency is modulated by the nature of working memory tasks. In the visual domain, Wutz et al. (2018) used magnetoencephalography (MEG) to demonstrate that alpha frequency responded to whether the task required temporal integration or segregation, that is to say, attention to periods of time or attention to very brief moments in time. In terms of alpha waves in tracking studies, Wutz et al. (2020) used MEG to show evidence of parietal alpha bursts during MOT. More of these bursts were observed when people were attempting to pay attention to individuated objects in the tracking display than when they were attempting to attend to them as a spatially grouped ensemble. These parietal alpha bursts seem likely linked with the inhibition related to suppressing attention to some objects in order to individuate others with attention. These results show that specifics of task instructions during tracking can modify the alpha response in terms of the amount of parietal alpha activity observed.

One might expect to see a relationship between an individual's peak alpha frequency at posterior sites and MOT tracking capabilities if indeed alpha cycles in the visual areas of the brain are responsible for discrete temporal sampling of the world. If this is true, for situations requiring continuous attention to highly dynamic stimuli, one might expect superior performance in people with faster posterior alpha rhythms, since this might allow them to sample the visual world more frequently and therefore to update their representations of moving stimuli more frequently, thereby increasing the spatiotemporal resolution of those representations. For this reason, Howard et al. (2017) conducted an EEG study and surprisingly showed that individuals with slower resting posterior alpha activity were better able to localize tracked objects. This result was in the opposite direction to that hypothesized, and the reasons for this are not known. One possibility considered by Howard et al. (2017) is that these effects may partially result from differences in function (e.g., with regard to working memory) between alpha oscillations in the upper and lower frequency bands. However, the position tracking task is different from a more typical MOT task, and there is recent evidence to highlight the opposite relationship between peak alpha frequency (PAF) and MOT performance. Zhang et al. (2021) explored how individual differences in occipital PAF predicted MOT performance among ice‐hockey players. They found that expert players relative to intermediate players had higher MOT performance but also that PAF positively predicted performance, which is what one might expect if higher PAF is related to more frequently updated representations of stimuli. Importantly, the findings of Zhang et al. (2021) also provide reasons to suggest that PAF may be a mediating factor between sporting expertise and MOT performance since sporting individuals had higher individual peak alpha frequency than non‐sporting individuals. There is some evidence around a possible link between sport and exercise and alpha frequency where some have argued that intense periods of physical exercise trigger an acute increase in PAF (Gutmann et al., 2018), for example, after engaging in endurance cycling (Di Fronso et al., 2019) and that tasks with greater demands might be more likely to induce these upwards shifts in alpha frequency, such as more demanding balance related tasks (Hülsdünker et al., 2016). The general aim of this research is to further explore the relationships between sporting expertise, PAF, and tracking performance.

We also have an opportunity to explore these relationships across different types of object tracking tasks. In the work previously mentioned, Mackenzie et al. (2022) specifically investigated the validity of a visuomotor‐controlled version of an object tracking task called the multiple object avoidance task (MOA) as a measure of visual attention function. This task required participants to track a number of objects on a screen while also controlling another object in a way to avoid colliding with the other objects. The authors provided evidence for its sensitivity in discriminating between sporting and non‐sporting expertise where those who engaged in sport outperformed those who did not. Although, again, the explanations for these differences were more general in nature. Although the attentional mechanisms are likely to be different in an active objective tracking task (active in the sense it required visuomotor control), the task still requires the frequent updating of spatial representations of moving stimuli. Therefore, one might also expect to see a positive relationship between an individual's posterior peak alpha frequency and performance in this task if (visual) alpha cycles are responsible for discrete temporal sampling. With this, although arguably more exploratory, we aimed to investigate whether the relationships between sporting expertise and PAF might explain performance in a more visuomotor active tracking task.

### 1.3 Aims and hypotheses

There were three main aims in this study.

1. We aimed to replicate previous research to identify if sporting expertise predicts MOT performance. We hypothesized that those who engaged in competitive sport would perform better than those who did not engage in sport. Including competitive sport as a criterion aids in the dissociation between experience and expertise where, typically, we observe cognitive differences between groups when there are different levels of expertise (Scharfen & Memmert, 2019).
2. We aimed to investigate if PAF predicts MOT performance. We hypothesized that higher PAF would relate to better performance. We also aimed to explore this in further tracking‐based tasks. Specifically, we aimed to investigate whether PAF predicts performance in the MOA task —an interactive MOT‐based task (Mackenzie et al., 2022) that has previously been demonstrated to be sensitive to sporting expertise. Although exploratory, given the similarity in the need to track multiple objects, we predicted that PAF would similarly predict MOA performance.
3. We aimed to investigate if PAF mediates the relationship between sporting expertise and MOT performance. We predicted that this would mediate this relationship where those who engaged in sport would elicit higher resting state PAF and that this would in turn positively predict superior MOT performance. We also aimed to explore this within the MOA task.

Using a correlational design, participants’ resting state (eyes closed) posterior PAF was measured and analyzed in relation to performance in two object tracking tasks. Given the theoretical focus of this study where we hypothesize a role of only occipital alpha, activities from only occipital electrodes were recorded. Given the theoretically driven rationale for the role of occiptial alpha, EEG recording from only occiptial areas was taken. This also removed an opportunity to retroactively cherry‐pick signals from other areas based on results (linked to principles of “hypothesising after results are known” [HARKing Kerr, 1998; Rubin, 2017]).

## 2 METHODS

### 2.1 Participants

Forty‐seven participants (30 females, 17 males) with a mean age of 28.4 (SD = 10.6) years took part in the study. A simple sample calculation was conducted in R using the package pwr (v.1.3‐0) which contains functions for basic power calculations using effect sizes and notations from Cohen (2013). A predicted effect size of Cohen *f* ^2^ = 0.28 (*R* ^2^ = ∼0.22) was indicated from the sport and MOT research discussed above that observed performance differences across sporting groups and relationships between PAF and sports with effect sizes ranging from *R* ^2 ^= 0.17 and *R* ^2 ^= 0.27 (Zhang et al., 2021). With an estimated power at 0.8, an alpha error probability of 0.05 in a simple general linear‐based model with four variables (where we assume three theoretical variables of interest and one covariate [age]), 47 participants would be required to achieve the predicted effect size. This group of participants was split into two groups. The sports group comprised 26 participants (12 females) with a mean age of 26.0 (*SD* = 7.95). They all had played sports competitively (for a club) for a minimum of 1 year. They were currently still competing/playing at the time of testing (see Table 1 for sporting expertise information). The non‐sport group had 21 participants (18 females) with a mean age of 31.4 (SD = 12.7). These participants declared they do not take part in competitive sport. Participants received an Amazon voucher worth £10 for participating and were recruited via internal advertising within Nottingham Trent University. Ethical permission was given to conduct the study by the Nottingham Trent University College of Research Ethics Committee (CREC; No. 2019/44).

**TABLE 1:** List of sporting expertise for sport group participants.

Participant	Sport type	Years playing competitively
DM_SP_1	Martial arts	6
DM_SP_2	Football	25
DM_SP_3	Gymnastics	12
DM_SP_6	Martial arts	5
DM_SP_7	Badminton	17
DM_SP_8	Triathlon competitive	3
DM_SP_9	Basketball	11
DM_SP_11	Running competitive	15
DM_SP_12	Canoe slalom	16
DM_SP_13	Cycling	20
DM_SP_18	Volleyball	6
DM_SP_25	Martial arts	5
DM_SP_27	Rugby	8
DM_SP_29	Running competitive	7
DM_SP_35	Hockey	14
DM_SP_36	Netball lacrosse	7
DM_SP_37	Squash	10
DM_SP_38	Triathlon competitive	32
DM_SP_39	Martial arts	4
DM_SP_40	BMX racing	15
DM_SP_41	Football	20
DM_SP_42	Football	10
DM_SP_43	Speed skating elite	18
DM_SP_44	Football	9
DM_SP_46	Rugby	7
DM_SP_47	Football	13

### 2.2 Materials

Both tasks below were presented on a 17.5‐inch CTX EX951F monitor (Chuntex Electronic Co., Ltd.) with a refresh rate of 85 Hz.

#### 2.2.1 Multiple object tracking task

**FIGURE 1:**
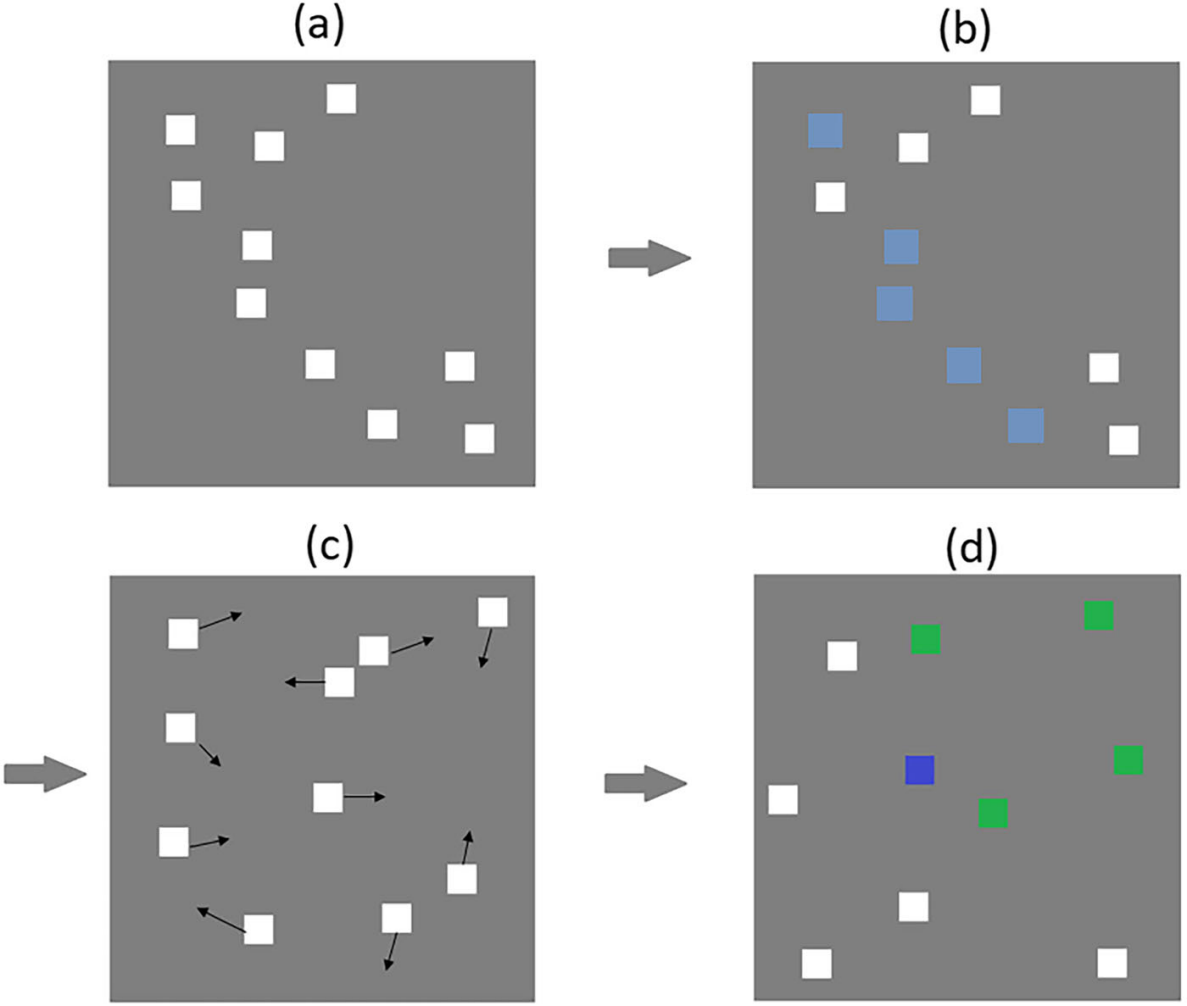
Static representation of the multiple object tracking (MOT) task. Participants viewed 10 white squares (a) before five of these squares would begin to flash (b). All squares would then move around the screen for a set period of time (c) before stopping. Participants then had to correctly identify the five squares that initially flashed (d). Feedback was given where green indicated correctly identified squares, and blue indicated incorrectly identified squares.

The MOT task was programmed using VisionEgg (Straw, 2008). Participants viewed 10 white squares (30 by 30 pixels, [∼7.9 by 7.9 mm]) on a gray background (1014 by 758 pixels). A randomly selected five squares then flashed for 3 s to indicate they were targets for tracking. All 10 white squares then moved randomly in a vector‐like fashion around the display. Each square moved in one of 12 randomly assigned angular directions (18, 45, 72, 108, 135, 162, 198, 225, 252, 288, 315, and 342 degrees) at a randomly assigned speed of either 60, 134, or 180 pixels per second. Squares could overlap with each other during this movement sequence. The squares then stopped. A square could not stop entirely on top of another square. It was the task of the participant to track the five target squares as all squares moved around. Each trial ended with the participant selecting (by way of a mouse click) the five squares they believed to be the targets (Figure 1). Participants completed 30 trials. The mean accuracy across the 30 trials was calculated as MOT performance. The number of trials used was comparable to previous MOT research (Howard & Holcombe, 2008; Howard et al., 2017; Mackenzie & Harris, 2017) where a reasonably large number of trials are required to obtain variability in ordinal accuracy data where participants can only get scores of 0%, 20%, 40%, 60%, 80%, and 100%, with most participants being able to achieve at least 60% (three correctly identified squares) in any given trial.

#### 2.2.2 Multiple object avoidance

**FIGURE 2:**
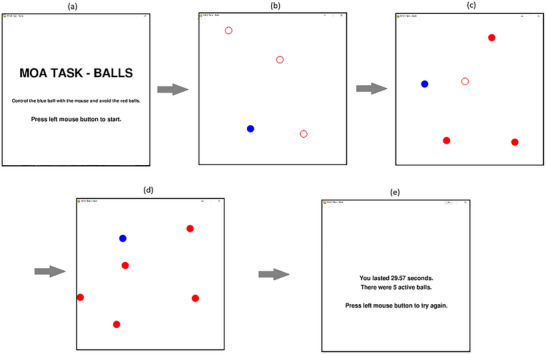
Static representation of the multiple object avoidance (MOA) task. Participants were presented with a start screen (a). After clicking the mouse button, participants were presented with a solid blue ball and three incomplete red balls (b). This was a period of preparation where participants could view the objects. After 2 s, the red balls would fill in, and all balls would begin to move. The participants had to avoid colliding the blue ball with the red balls. After 10 s, another red ball was added where it was first presented without fill and could not be collided with for 2 s (c). This pattern continued (d) until the participants could no longer avoid a collision (e).

We wished to extend our predictions by exploring the role PAF might play in not only standard MOT tasks but also other tracking‐based tasks. We previously developed and validated an interactive tracking task called the MOA task (Mackenzie et al., 2022). In the MOA task, three (red) obstacle balls were presented on the screen alongside a single (blue) ball. The participant controlled the position of the blue ball with a mouse. The red balls began to move around the screen, with a random initial direction conforming to typical straight‐line vector‐physics. The participants’ task was to avoid the blue ball colliding with the red balls by moving the blue ball with the mouse. After 10 s, one more red ball was added to the visual display. This pattern continued until the participant was unable to avoid a collision and the trial ends (Figure 2). Participants completed 10 trials (2 practice trials), and the length of time for each trial was taken as a measure of performance. A longer trial length indicates better performance. An average across eight trials was recorded as performance. Ball sizes were each 40 pixels in diameter (∼10.6 mm), and speeds ranged from 0 to 680 pixels per second. The task window is displayed at a size of 800 by 800 pixels.

### 2.3 EEG data acquisition and signal processing

Participants sat in a comfortable chair ∼60 cm away from the computer monitor and were instructed to sit as still as possible for the duration of the recording. Following the application of the EEG cap (64‐channel Biosemi headcap), a conductive gel (Signa gel) was inserted into electrode inlets. Participants completed an eyes‐closed recording lasting 2 min. Using recordings from when eyes are closed is consistent with previous research (Howard et al., 2017; Zhang et al., 2021) where data would be largely noise‐driven due to ongoing visual stimulation when recording when eyes are open. Two minutes were deemed an appropriate resting period to derive resting state alpha given previous literature (e.g., Gutmann et al., 2015; Lum et al., 2022; Wu et al., 2014) where increasing the duration would not offer increased precision in deriving PAF, and a short duration allows participants to remain focused and affords less opportunity for movement artifacts in the data.

Continuous EEG was recorded using a Biosemi ActiveTwo system (Amsterdam, the Netherlands) at 2048 Hz and digitized with 24‐bit resolution. Following the application of the electrodes, extra gel was added and hair parted (if applicable) if the impedance was above 5 kΩ. Event triggers indicating the start and end of the block were sent to the EEG amplifier via a USB to TTL box (Neurospec AG).

Data were re‐referenced offline to Cz and down‐sampled to 512 Hz, and a 0.5‐Hz high‐pass (3381 points, −6 dB at 0.25 Hz) filter and a 40‐Hz low‐pass (171 point, −6 dB at 45 Hz) filter were applied using a zero‐phase, non‐causal linear finite impulse response filter. After filtering, data were visually inspected for bad channels and periods of residual high‐frequency noise. Given that we recorded from only three occipital channels (and Cz), it was not possible to spherically interpolate bad channels. As such, the visual inspection of online EEG ensured that each participant had low impedance at each electrode and that the signal was of sufficient quality. If any channel became noisy during the recording, the recording was stopped and repeated, following the application of more gel. As such, no bad channels were present in the final data for all participants. Any periods of data that contained residual high‐frequency noise (such as those induced by movement) were manually removed. Finally, data were epoched from trial onset to 105 s. We removed the first 15 s of data as we speculated that participants could still be settling in to the task, and thus this period would not reflect a resting state. EEG data were imported and processed using functions from the EEGLAB (v2019.1) environment (Delorme & Makeig, 2004) for MATLAB (The Mathworks, Inc.).

#### 2.3.1 Peak alpha frequency quantification

In order to derive peak alpha frequency, power spectral density estimates of the epoched EEG data were achieved using the spectopo EEGlab function (frequency factor = 8). This resulted in mean log power estimates between DC and the nyquist frequency (256 Hz). The derived power values were then averaged across three occipital electrodes (O1, Oz, and O2), and the frequency between 7 and 14 Hz that had the highest value was taken as peak alpha frequency.

### 2.4 Procedure

Participants completed both tracking tasks and the EEG recording procedures (as above) in one sitting. The order of the tasks and the order of when the EEG recording (either before or after the tasks) were counterbalanced across all participants to account for typical alertness and fatigue effects across our participants. Breaks were given between tasks and recordings. For the MOT task, participants were asked to attend to the flashing squares that would appear on the screen and maintain attention toward these same squares as all squares moved around. Thirty trials were completed. For the MOA, participants were instructed they would be controlling the blue ball on screen and must avoid the moving red ball and were informed that the task would increase in complexity with more balls being added the longer they maintained a collision‐free trial. Ten trials were completed (two practice trials and eight experimental trials). Participants also completed a short questionnaire asking about their sporting experience. These questions included: Do you play sports/a sport, please list which sport(s), at what level (e.g., nationally, club‐level, recreationally), what is your main sport and how often do you engage in this sport?

### 2.5 Statistical design

Correlations were initially conducted to determine relationships between Sporting Group, PAF, and task performance (point‐biserial correlations were used for the sporting groups as this variable is categorical). General multivariate linear models were conducted to determine the relationships between (1) sporting group and task performance and (2) PAF and task performance. Mediation analyses were conducted to model the relationship between the predictor (sporting group), the outcome (task performance), and the mediator (PAF). All analyses were conducted in R version 4.2.1. Mediation models were fitted using the R package *mediation* version 4.5.0 (Tingley et al., 2019), and bootstrapping principles outlined by Preacher and Hayes (2004) were applied where the indirect mediation path was computed, sampling with replacement, across 1000 bootstrap samples. There was no significant difference in age between the sporting and non‐sporting groups (*t* (45) = 1.78, *p =* .08); however, given that alpha typically slows with age (e.g., Stacey et al., 2021), it will still likely explain some variability in these models and therefore should be accounted for as a covariate to reduce type 1 error probability.

## 3 RESULTS

All data and R scripts are available on the OSF (https://osf.io/9ch2u/?view_only = 1c0bcd3eac114224a00bdfa9bbb354d7).

The performance of the MOT task was calculated as a percentage of correctly identified squares (out of five). This was averaged across the 30 trials. Given that data are in percentages, these data were transformed using a logit function for all general linear‐based analyses. The performance of the MOA task was calculated as the time (in seconds) each trial lasted. This was averaged across eight experimental trials. Descriptive statistics and correlations can be viewed in Table 2.

**TABLE 2:** Descriptive statistics and correlations (*r* values) of task performance and peak alpha frequency (PAF) by sporting grouping. Multiple object tracking (MOT) performance is measured as a percentage, and multiple object avoidance (MOA) performance is measured in seconds.

Descriptive statistics							Correlations			
Group	Measure	*N*	Min	Max	Mean	SD		MOT	MOA	PAF
Sports	MOT	26	68.0	91.3	81.1	6.7	MOT	–		
	MOA	26	10.7	52.1	25.6	8.88	MOA	0.32^*^	–	
	PAF	25	7.75	12.4	10.3	0.97				
Non‐sports	MOT	21	61.3	94	73.5	9.05	PAF	0.43^**^	0.14	–
	MOA	21	11.0	32.4	19.0	6.49	Sporting group	0.40^**^	0.39^**^	0.35^*^
	PAF	21	7.12	13.9	9.43	1.52				

*Note*: Positive values denote positive relationships where better performance correlates to better performance (between tasks), higher PAF, and/or for the dichotomous sporting grouping, with those who participate in sport. One participant in the sporting group was unable to provide a stable EEG reading and is therefore included only in behavioral analyses).

Abbreviations: N, number of participants; Min, minimum; Max, maximum; SD, standard deviation.

*p < .05; **p < .01; ***p < .001.

### 3.1 Aim 1: Does sporting expertise predict tracking task performance?

**FIGURE 3:**
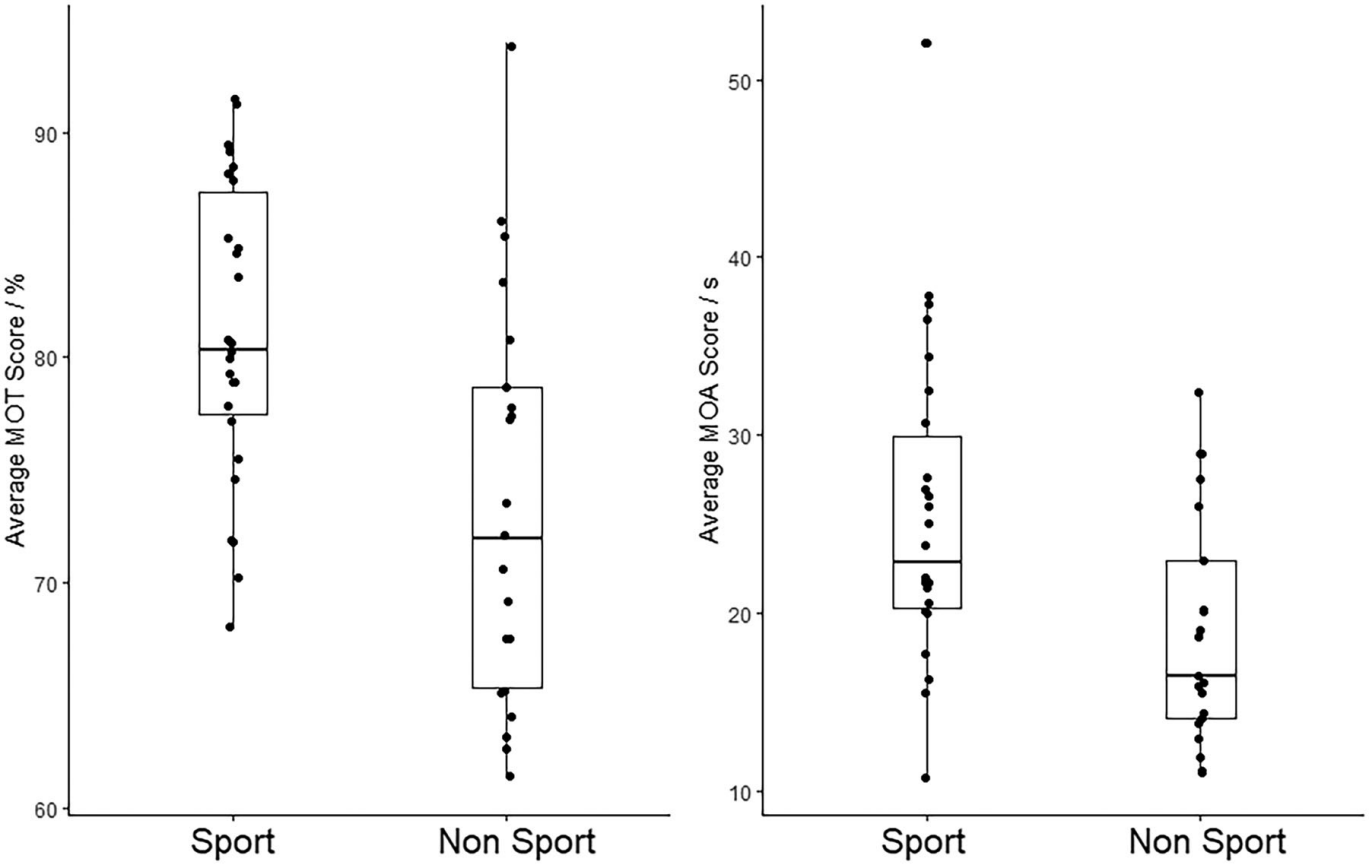
Differences in task performance between sporting groups. Data show ranges, medians, and interquartile ranges.

Task performance as predicted by the sporting group was modeled in a multivariate linear model. Age was included as a covariate throughout. The multivariate model revealed that the sporting group predicted task performance where those in the sports group outperformed those in the non‐sports group (*V *= 0.19, *F*(2,43) = 5.16, *p* = .01) overall. For the MOT task, those in the sports group performed better than those in the non‐sporting group (*β* = 0.36, SE = 0.15, *t* = 2.52, *p =* .015), and similarly for the MOA task, those in the sports group performed better than those in the non‐sporting group (*β* = 0.35, SE = 2.39, *t* = 2.46, *p =* .02) (Figure 3).

### 3.2 Aim 2: Does PAF predict MOT and MOA performance?

**FIGURE 4:**
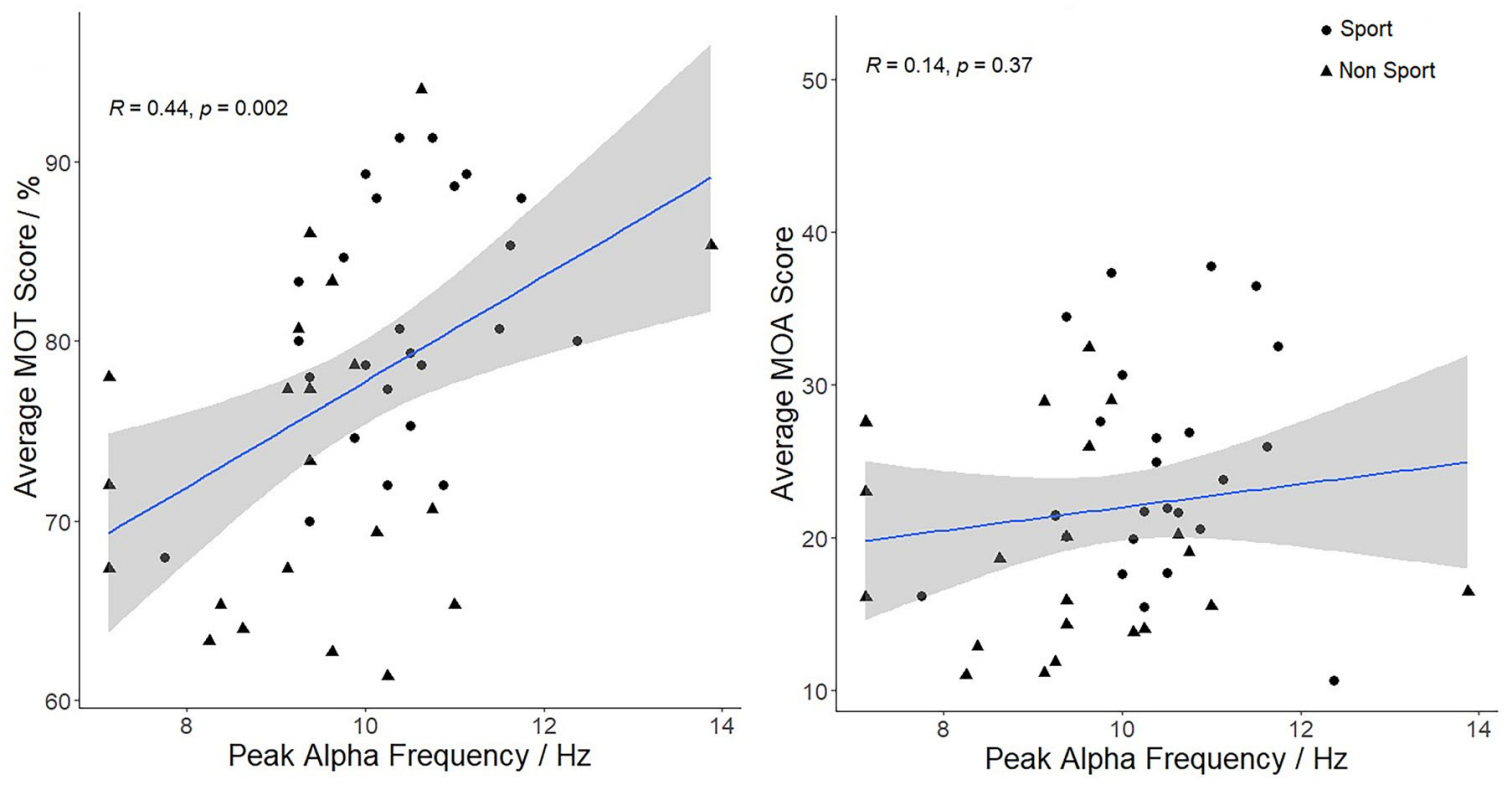
Relationships between peak alpha frequency (PAF) (Hz) and task performance.

PAF was modeled as a predictor of task performance in a multivariate general linear model (with age as a covariate). The multivariate model revealed that PAF predicted task performance, where higher PAF predicted better task performance (*V *= 0.16, *F*(2,42) = 4.11, *p =* .02) overall. This was driven by the MOT task, where higher PAF predicted better MOT performance (*β* = 0.40, SE = 0.06, *t* = 2.89, *p =* .006) but did not predict MOA performance (*β* = 0.07, SE = 0.10, *t* = 0.50, *p =* .62) (Figure 4).

### 3.3 Aim 3: Does PAF mediate the relationship between sporting expertise and MOT performance?

**FIGURE 5:**
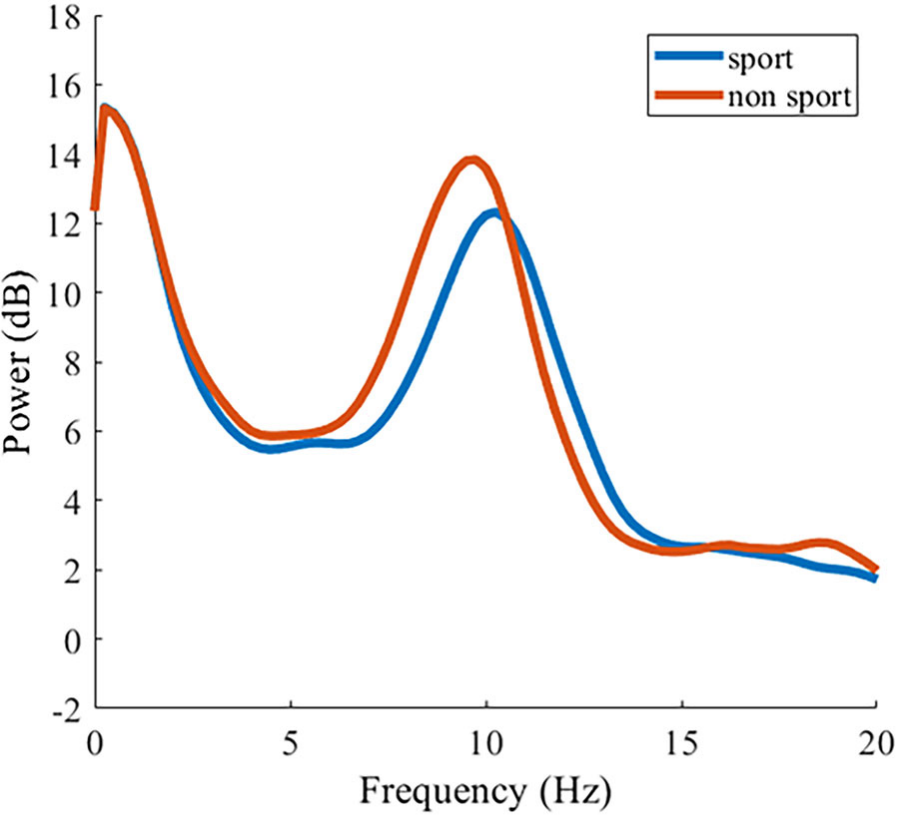
Grand average spectra plots showing peak alpha frequency (PAF) difference between sporting and non‐sporting groups (power is decibel normalized at 10xlog10 (μV2/Hz). Note: there is no difference in power at PAF level between the groups (*p* = .26).

Correlations between MOT, MOA, PAF, and the sporting group can be viewed in Table 2 and are all significant except for the correlation between PAF and MOA. Importantly, we observe that PAF in the sporting group is higher than the non‐sporting group (Table 2; Figure 5). These data were included in a mediation model with the sporting group predicting task performance, mediated by PAF. There was no correlational evidence that PAF would predict MOA performance, and therefore these relationships were not modeled further.

Mediation analyses were conducted using the R package *mediation* (Tingley et al., 2019). Addressing power limitations and issues with non‐normality, bootstrapping principles outlined by Preacher and Hayes (2004) were applied, and the indirect mediation path (*ab*) was computed, sampling with replacement, across 1000 bootstrap samples. The results of the mediation analyses can be viewed in Table 3. Age was included as a covariate.

**TABLE 3:** Summary of mediation model.

	Standardized estimate	95% CI (lower)	95% CI (upper)	*p*‐value
Average direct effect (sports → MOT performance)	−0.27	−0.64	0.12	.16
Average mediation effect (indirect path: sporting group > PAF > MOT performance)	−0.11	−0.30	0.00	.042
Total effects	−0.38	−0.70	−0.02	.040

Abbreviations: CI, confidence interval; MOT, multiple object tracking; PAF, peak alpha frequency.

The overall mediation model was significant (*p =* .04). There was a significant mediation effect of PAF on the relationship between the sporting group and MOT performance (*p =* .042). Interestingly, the direct effect between the sporting group and MOT performance in the model was not significant (*p =* .16).

## 4 DISCUSSION

The aims of this study were threefold. The first was to replicate previous findings that those who engage in sporting activity outperform those who do not engage in attention tasks, specifically object tracking tasks. We find this here for both the MOT and MOA tasks. The second aim was to explore whether resting state posterior individual peak alpha frequency predicted performance in tracking tasks. We found that higher PAF predicted better MOT performance but not MOA performance. The third aim was to explore PAF as a possible mediating factor in the relationship between sporting expertise and MOT performance. We found some evidence that PAF may mediate this relationship where those who engage in sport displayed higher PAF and in turn performed better in the MOT task.

Sporting expertise often predicts performance in attention tasks where one often observes a superiority effect in either those who play sport compared to those who do not, or within a sport where increased expertise predicts performance (Harris et al., 2020; Howard et al., 2018; Memmert, 2006; Qiu et al., 2018). One might explain these performance differences more generally through cognitive transfer or related deliberate practice type explanations (Dahlin et al., 2008; Ericsson et al., 1993). If one assumes similar types of attentional processes in these attention tasks as in sporting activity such as object tracking, dividing attention, and sustaining attention, one might predict training of these processes is occurring when engaging in sport. The current study however may offer some further understanding toward these links between sports and attention performance.

As described in the theory of perceptual cycles, at posterior sites where visual processing occurs, it has been argued that alpha oscillations reflect discrete temporal sampling in vision (Dugué et al., 2011; VanRullen, 2016) whereby periodic phases of visual inhibition are followed by periods of visual sampling. As proposed by Howard et al. (2017), one might expect therefore to see a relationship between an individual's peak alpha frequency and tracking capabilities if faster discrete temporal sampling of the world assists with updating position representations during tracking. We find evidence for this prediction here where PAF predicted MOT performance, and we also thereby replicate the results of Zhang et al. (2021). These results provide further support for the perceptual cycles theory of visual attention. Importantly, in the mediation analysis, the direct relationship between sporting expertise and performance disappeared suggesting that higher PAF in sporting individuals explains MOT performance and that it is not just some kind of cognitive skill transfer from sport that relates to superior attention performance. In other words, performance was driven by PAF and not simply because of playing sport.

However, it is not yet clear why Howard et al. (2017) found the opposite pattern of results for the relationship between PAF and tracking ability: in that study, slower PAF was associated with better performance, indicated by higher precision position estimates during a variant of the MOT task where targets were not intermingled with distractors. In that study, the task was not to identify which objects were targets, but rather to report their final position before disappearance as accurately as possible. This emphasis on high precision position estimates rather than on attending to which objects were targets amongst distractors seems a likely cause of the difference in findings between results. One further possibility is that the relationship between PAF and tracking depends on the extent to which the task engages dorsal frontoparietal attention networks (as contrasted with ventral processes, see Tosoni et al. (2023) for a recent discussion). Since MOT is traditionally considered to be a measure of spatial attention, dorsal stream activity during tracking has been implicated and confirmed by neuroimaging studies (Alnæs et al., 2015; Atmaca et al., 2013; Merkel et al., 2015). One might argue that the position report task used by Howard et al. (2017) may have relied less on these dorsal processes than the MOT task in the current study and more on ventral stream processes related, for example, to pattern recognition. If this were the case, it could potentially explain the different pattern of results found here and in the previous work of Howard et al. (2017). Future studies might directly investigate the extent to which relationships between tracking performance and PAF relate to dorsal and ventral processes.

Interestingly, we do not observe the relationship between PAF and performance on the MOA task. It seems unlikely that differences in dorsal network recruitment could explain the different results reported here for MOT and MOA, given that dorsal involvement is likely implicated in both these tasks. Although the ability to track objects is important in both, the MOA task is much more active in nature in the sense it requires visuomotor control. It may then be that the various aspects of visuomotor control required in the MOA task, such as motor planning, predicting near future locations of objects and deciding where to move the cursor, may not be related to PAF and likely contributed other sources of variability to the MOA score.

The finding that those with sporting expertise displayed higher PAF replicates recent work (Zhang et al., 2021). However, evidence regarding a potentially causal role for sport in PAF is generally mixed. Some have argued that intense periods of physical exercise or greater task demands trigger an acute increase in PAF (Gutmann et al., 2018; Hülsdünker et al., 2016). Others do not replicate these kinds of effects, with Christie et al. (2017) reporting no shift in IAPF after an ice hockey shooting task who also reported no relationship between alpha frequency and performance on this task. In terms of a correlation between a person's physical activity levels and PAF, there are similarly mixed results: De Frutos‐Lucas et al. (2018) found such a relationship for older adults using MEG, whereby those reporting more physical activity had higher PAF. However, Hülsdünker and Mierau (2021) found no differences between athletes and non‐athletes in terms of PAF. It is therefore not currently clear if engaging in sport can modify PAF, and what the mechanism for this would be. Although there is some evidence to suggest acute bouts of sporting activity may influence alpha, the longer term effects of sporting activity are not currently understood, partly due to the difficulty of carrying out longitudinal studies on this topic. It is also unclear whether engaging in sport itself or engaging in, more generally, intense cardiovascular fitness that may influence alpha. Further, one might propose the opposite relationship is equally possible where individuals who generally have higher alpha may be more likely to be better at sport which could encourage participation and continuation in a sport, rather than alpha changing due to the engagement in sport. In addition, in the current study, the definition of sports was very wide, including sports that differed in nature, from triathletes to football (soccer) players. This was a purposeful grouping for both logistical reasons (to maximize a sample) and theoretical reasons where there was evidence to suggest that engagement in even more passive sports (e.g., golf) may influence alpha (e.g., Gallicchio et al., 2017). Future research would benefit finer grained grouping of sport categories, for example, interceptive/strategic versus passive sport (e.g., running) versus sedentary. This would provide more fidelity in whether it is a specific sport type driving the higher PAF but also tap into the question of whether it is simply exercise that might drive this. These are all exciting future questions, likely answered more longitudinally, that will advance the field.

## 5 CONCLUSIONS

Higher posterior PAFs were observed in individuals who engaged in sporting competitively, and this in turn predicted multiple object tracking performance. It is possible that higher PAF is one mechanism by which sporting individuals appear to possess some superiority in attentional and perceptual tasks. Future research should address whether this higher PAF plays a causal role, for example, by way of a training intervention. The relationship between PAF and MOT performance may be due to more frequent perceptual sampling in the presence of higher posterior alpha activity, whereby individuals update their representations of spatial locations of objects more frequently.

## AUTHOR CONTRIBUTIONS

**Andrew Mackenzie**: Conceptualization; data curation; formal analysis; funding acquisition; investigation; methodology; project administration; supervision; visualization; writing—original draft; writing—review and editing. **Joshua Baker**: Data curation; formal analysis; investigation; methodology; visualization; writing—original draft; writing—review and editing. **Rosie Daly**: Data curation; investigation; methodology. **Christina Howard**: Conceptualization; investigation; writing—original draft; writing—review and editing.

## CONFLICT OF INTEREST STATEMENT

The authors declare no conflicts of interest.

### PEER REVIEW

The peer review history for this article is available at https://publons.com/publon/10.1002/brb3.3434.

## Data Availability

All data and R scripts used in the study are available on the OSF (https://osf.io/9ch2u/?view_only=1c0bcd3eac114224a00bdfa9bbb354d7).
